# Supplementary material for: Socioeconomic predictors of COVID-19-related health disparities among United States workers: A structural equation modeling study
Source: PLOS Glob Public Health. 2022 Feb 9;2(2):e0000117. doi: 10.1371/journal.pgph.0000117 (PMC10021756; doi:10.1371/journal.pgph.0000117)
Supplement: Attachment — Submitted filename: Response_to_reviewers_PGPH_FINAL.docx [file pgph.0000117.s004.docx]

PGPH-D-21-00803
Socioeconomic predictors of COVID-19-related health disparities among U.S. workers: A structural equation modeling study
PLOS Global Public Health

Dear Dr. Cowgill,

We are grateful for the opportunity to resubmit the manuscript, entitled “Socioeconomic predictors of COVID-19-related health disparities among U.S. workers: A structural equation modeling study” and thank you and the reviewers’ thoughtful comments.

Sincerely,

Yesim Tozan, PhD

**Response to Editor**

1. From [https://journals.plos.org/globalpublichealth/s/submission-guidelines#loc-methods-software-databases-and-tools](https://urldefense.proofpoint.com/v2/url?u=https-3A__journals.plos.org_globalpublichealth_s_submission-2Dguidelines-23loc-2Dmethods-2Dsoftware-2Ddatabases-2Dand-2Dtools&d=DwMGaQ&c=slrrB7dE8n7gBJbeO0g-IQ&r=XGzUN6S7CV-sOjf7f4TPQQ&m=E9QPnFyiyBoRefLDCmTao2ISpcpvaoIH1f0iiaaQtnxk4mnM42kAfubXGQ8e0LSO&s=yUcmcSAUwrRFDpjb08pj1ERI634wBQ6nLZQ2O8gUtKI&e=) : Submissions to PLOS GPH presenting methods, software, databases, or tools (including models) must demonstrate that the new tool achieves its intended purpose. This requirement may be met by including a proof-of-principle experiment or analysis; if this is not possible, a discussion of the possible applications and some preliminary analysis may be sufficient.
   a. Would you please discuss how you might validate this model and what its possible applications are?

**RESPONSE:** One of the expectations of statistical models for use in the field of public health is for the model’s utility to extend beyond the data employed to develop and test it. This process often entails the development of a theory-based model using one sample and then validating the model in another sample (Cliff, 1983). A structural equation model tests a series of hypotheses related to the covariance structures that underlie the relationship between variables (Browne & Cudeck, 1989), which would ideally be cross-validated in other samples by testing the model with other data. However, in cases when such data are not available, it is an acceptable practice to test whether the covariance structure, and by extension, the findings, hold true for split random samples (Browne & Cudeck, 1989). To test this, as a post hoc analysis, we split the sample in halves randomly. We present the post hoc analysis findings in two additional tables (Tables S2 and S3 in the supplementary material.) We found that both the model fit and the estimated coefficients were similar in the full and split samples. Specifically, we found that the model fit the data equally well in both split samples; in terms of the coefficients, none of the coefficients changed signs in the split samples with respect to the full sample, and only in 4 estimates, the significance changed. Thus, we can conclude that the model is robust.

We added the following text (lines 205-2011) with two new references:

One of the expectations of statistical models for use in the field of public health is for a model’s utility to extend beyond the data employed to develop and test it [41]. This process often entails the development of a theory-based model using one sample and then validating the model in another sample [42]. However, this is at times not possible. To test the robustness of our model, we randomly split the sample in halves and assessed if the data fit the model adequately in each subsample, and if the direction and significance of the coefficients remained stable [42].

And a new section describing the post hoc analyses in the Results section (lines 293-298)

We found evidence for the robustness of the model as both the model fit and the estimated coefficients were similar in the full and random split samples. The model fit the data equally well in the full and split samples (Table S2), the signs of the coefficients in both split samples were consistent with those of the full model, and the significance level changed in only 4 of the 36 coefficients estimated (Table S3).

Cliff, N. (1983). Some cautions concerning the application of causal modeling methods. Multivariate Behavioral Research, 18(1), 115–126. <https://doi.org/10.1207/s15327906mbr1801_7>

M.W. Browne & R. Cudeck (1989) Single Sample Cross-Validation Indices for Covariance Structures, Multivariate Behavioral Research, 24:4, 445-455, DOI: 10.1207/s15327906mbr2404_4

2. 58-69: please provide references for these assertions

**RESPONSE:** References numbers 13 to 19 (new numbering) were added, as requested.

3. 179: what was the cutoff for determining satisfactory fit using CFI/RMSEA/SRMR?

**RESPONSE:** The cutoff criteria and appropriate reference were added as follows in lines 200-202:

CFI values of ≥ 0.95, RMSEA values ≤ 0.06 and SRMR values ≤ 0.08 were considered to determine appropriate model fit [40].

40.	Hu Lt, Bentler PM. Cutoff criteria for fit indexes in covariance structure analysis: Conventional criteria versus new alternatives. Structural Equation Modeling: A Multidisciplinary Journal. 1999;6(1):1-55.

4. 206-208: this is a bit confusing – which factors are you referring to here? please clarify if it was 15% (as stated in the abstract) or 19% (as stated here) of respondents who experienced three factors; also please clarify the characterization of 19% as a majority and which 5 factors you’re referring to here given that in the abstract you refer to three factors. Or are you referring here to the characteristics listed in Table 1? Later you use the term “vulnerability” instead of “factor”.

**RESPONSE:** We agree that this point was not made sufficiently clear. In the paper, we make the distinction between factors, which are single items, and vulnerabilities, which entail a cluster of factors. This is explained in lines 102-105, to which we added the sentence in italics to clarify this point: For the purposes of this study, we define vulnerability as a cluster of closely-correlated factors that place specific population subgroups at greater risk for adverse COVID-19-associated outcomes. *We consider eleven socioeconomic factors in the model.*

We entered 11 individual factors in the SEM model to assess clustering, which are listed in Table 1: 1) Annual household income, 2) No fixed income, 3) Lost income due to COVID-19, 4) Is food insecure, 5) Can’t afford to quarantine, 6) Can’t work from home, 7) Depressive symptoms, 8) Anxiety symptoms, 9) Stress symptoms, 10) Employer does not offer paid sick leave, and 11) No health insurance. We made changes in the terminology used in the table to further distinguish the factors from the vulnerabilities. In the SEM, the factors clustered into three vulnerabilities (financial, mental health, and healthcare access); 15% of respondents experienced the three vulnerabilities (This shown on Table 3).

We also added the following clarification to the methods (additions in italics, line 182):

After examining the distribution and frequencies of the variables of interest, we used structural equation modeling (SEM) to evaluate a hypothesized conceptual model of COVID-19-related vulnerabilities and *to assess clustering of eleven socioeconomic factors* associated with these vulnerabilities (Fig 1).

In the results, we added a Figure [Figure 2]:

Over a quarter of respondents did not have paid sick leave (27.1%), and 4.9% did not have health insurance. When examining the cumulative distribution of factors, a majority of respondents experienced 3 factors (19%), with most respondents endorsing between 1 (13%) and 5 (12%) factors *(Fig 2).*

5. 330-331: can you be more specific about where or which structural inequalities are discussed in the paper vis-à-vis BIPOC specifically (most discussion in the Introduction is about inequality by sex and SES)?

**RESPONSE:** This paper addresses two types of structural inequalities: 1) precarious employment conditions (e.g., such as having low and not fixed income, which in turn is associated with lack of basic needs (e.g., food insecurity), and 2) limited access to care (e.g., no health insurance and no paid sick leave). You are correct that these structural inequalities are not discussed vis-à-vis BIPOC, but rather, in general, with a specific focus on sex, urban/rural residence, and essential worker status.

To clarify this point we added the following text in italics and the references listed below (lines 374-378:

“In particular, BIPOC are less likely to have access to broadband and social media than their white counterparts *since they are more likely to experience some of the structural inequalities addressed in this paper, such as precarious employment conditions and unmet basic needs [ATSKE].* In addition, BIPOC and those with poor mental health may be more reluctant to participate in web-based surveys [Watson; Jang; Center for Behavioral Statistics and Quality].”

1. Atske S, Perrin A. Home broadband adoption, computer ownership vary by race, ethnicity in the U.S. 2021; <https://www.pewresearch.org/fact-tank/2021/07/16/home-broadband-adoption-computer-ownership-vary-by-race-ethnicity-in-the-u-s/.> Accessed August 10, 2021.
2. Watson B, Robinson DHZ, Harker L, Arriola KRJ. The Inclusion of African-American Study Participants in Web-Based Research Studies: Viewpoint. *Journal of medical Internet research.* 2016;18(6):e168-e168.
3. Jang M, Vorderstrasse A. Socioeconomic Status and Racial or Ethnic Differences in Participation: Web-Based Survey. *JMIR Res Protoc.* 2019;8(4):e11865-e11865.
4. Center for Behavioral Health Statistics and Quality. *Nonresponse Bias in Demographic and Survey Estimates from the Mental Health Surveillance Study of the National Survey on Drug Use and Health.* Rockville, MD: Substance Abuse and Mental Health Services Administration;2017.
6. 338-9: is this a realistic recommendation at this point if not already carried out?

**RESPONSE:** We agree with this point and have modified the recommendation as follows (line 386):

For policymakers to accurately measure impact of COVID-19 on socioeconomic vulnerabilities, research is warranted utilizing a nationally representative sample.

**Journal Requirements:**

1. Please provide separate figure files in .tif or .eps format only, and remove any figures embedded in your manuscript file.

**RESPONSE:** The images have been attached as .tif files.

2. Please note that your Data Availability Statement is currently missing the DOI/accession number of each dataset OR a direct link to access each database. If your manuscript is accepted for publication, you will be asked to provide these details on a very short timeline. We therefore suggest that you provide this information now, though we will not hold up the peer review process if you are unable.

**RESPONSE:** The data are available here: <https://www.openicpsr.org/openicpsr/project/120308/version/V1/view;jsessionid=29ED0C50EF80411A6C5399451819AA08>

Please review your reference list to ensure that it is complete and correct. If you have cited papers that have been retracted, please include the rationale for doing so in the manuscript text, or remove these references and replace them with relevant current references. Any changes to the reference list should be mentioned in the rebuttal letter that accompanies your revised manuscript. If you need to cite a retracted article, indicate the article’s retracted status in the References list and also include a citation and full reference for the retraction notice.

**RESPONSE:** We have not included any retracted articles. However, we have added new citations (listed below) in response to some of the clarifications requested by the reviewers.

Cliff, N. (1983). Some cautions concerning the application of causal modeling methods. Multivariate Behavioral Research, 18(1), 115–126. <https://doi.org/10.1207/s15327906mbr1801_7>

Browne, MW & R. Cudeck (1989) Single Sample Cross-Validation Indices for Covariance Structures, Multivariate Behavioral Research, 24:4, 445-455, DOI: 10.1207/s15327906mbr2404_4

Hu Lt, Bentler PM. Cutoff criteria for fit indexes in covariance structure analysis: Conventional criteria versus new alternatives. Structural Equation Modeling: A Multidisciplinary Journal. 1999;6(1):1-55.

Atske S, Perrin A. Home broadband adoption, computer ownership vary by race, ethnicity in the U.S. 2021; <https://www.pewresearch.org/fact-tank/2021/07/16/home-broadband-adoption-computer-ownership-vary-by-race-ethnicity-in-the-u-s/.> Accessed August 10, 2021.

Watson B, Robinson DHZ, Harker L, Arriola KRJ. The Inclusion of African-American Study Participants in Web-Based Research Studies: Viewpoint. *Journal of medical Internet research.* 2016;18(6):e168-e168.

Jang M, Vorderstrasse A. Socioeconomic Status and Racial or Ethnic Differences in Participation: Web-Based Survey. *JMIR Res Protoc.* 2019;8(4):e11865-e11865.

Center for Behavioral Health Statistics and Quality. *Nonresponse Bias in Demographic and Survey Estimates from the Mental Health Surveillance Study of the National Survey on Drug Use and Health.* Rockville, MD: Substance Abuse and Mental Health Services Administration;2017.

Haley D, Paucar-Caceres A, Schlindwein S. A Critical Inquiry into the Value of Systems Thinking in the Time of COVID-19 Crisis. *Systems.* 2021;9(1).

Strelkovskii N, Rovenskaya E. Causal Loop Diagramming of Socioeconomic Impacts of COVID-19: State-of-the-Art, Gaps and Good Practices. *Systems.* 2021;9(3).

Gostin LO, Wiley LF. Governmental Public Health Powers During the COVID-19 Pandemic: Stay-at-home Orders, Business Closures, and Travel Restrictions. *JAMA.* 2020;323(21):2137-2138.

Additional Editor Comments:


Title: write out U.S.
32: replace comma with a colon
33: please define beta and B in the abstract
37: as Reviewer 2 pointed out, mental health results are missing from the abstract
42: write out “coronavirus disease” prior to using the abbreviation COVID for the first time
43: delete comma, delete hyphen
45: please rewrite this sentence to avoid potential confusion – as written, “It” could be interpreted as referring to the survey rather than the pandemic
47: suggest specifying COVID-19 exposure risk
53 and 55 and 73 elsewhere: delete “etc.”
54: write out SARS-CoV-2 full name before using the abbreviation
77: insert hyphen: “calls for a systems-thinking approach”
81: insert hyphen: “testing-site deployment”; delete comma
82: if there are any such studies, please cite them here; if there are none that you’re aware of, modify the sentence to state that
86: delete hyphen
87: insert hyphen: “adverse COVID-19-associated outcomes”
91: delete colon
92: delete comma
105: delete colon
107: please include the name of the institution in the revised submission (note that this review is single-blind and so the authors’ names and institutions are already visible to reviewers)
114: delete colon; change semicolon following “18-39 years” to comma
115-6: change square brackets to parentheses and write out BIPOC
116-118, 124: replace semicolons in parentheses with commas
124-5 and 142: add space on either side of > or <
128: write out USDA
128-9, 182: add space on either side of = sign
131: add comma after “question”
135: delete comma
150: delete hyphen
153: delete colon and semicolon
Figures 1 and 2 captions: please add “among U.S. workers”
183: suggest stating “the model’s sample size was reduced from 2845 to 2800”
190: add comma after “Table 1”
196: delete hyphen
197-8: add hyphen “food-insecure”
200-201: remind the reader what the total possible score was (i.e., 1.7 on a scale of 6)
205: delete comma
226: “mental health”
Table 2 and Table 3 title: add population, N, date as in Table 1
243: replace “less” with “fewer”
252: add -d: “experienced”
254-5: keep semicolon after “vulnerabilities” but replace other semicolons with commas
259: add comma after “mental health”, hyphenate “systems-thinking” here where it modifies “approach”
268: hyphenate “safety-net”; add “leave” after “paid sick”
270: comma after “wages”
277: insert “those with” after “including”
278: hyphenate “access-to-care barriers”
281: delete comma
287: replace “than” with “of”
299: it may be helpful to remind readers that this was only a few weeks after the first pandemic restrictions were imposed in the US (also at line 102)
301: move “only” – “was observed only in urban centers”
306: hyphenate “healthcare-access barriers”
308: delete “the” before “lower mental health”
310-312: it may be helpful to provide more context here about the extent and timing of restrictions (in most cases these were not true lockdowns)
316: hyphenate “systems-thinking approach”
320: hyphenate “convenience-sampling strategy”
322: change “importantly” to “important”
330-1: comma after “counterparts”
342: hyphenate “systems-thinking approach”
345-7: suggest rewording these two sentences – perhaps “As the US prepares for post-pandemic recovery, it should put equitable resource allocation to support the varying needs of the population at the core of its public policy agenda. To do this, policymakers must …”
349: delete hyphen
References: please capitalize COVID and proper nouns (e.g., United States) in the reference list


**RESPONSE:** Thank you for this thorough review. We applied all the changes requested, with the exception of removing the square brackets on line 115-116 because we had (BIPOC) inside them (new lines 136-137).

**Reviewers' comments:**

Reviewer #1: The theme is relevant and current. The study is well conducted and has practical application.
The authors present the research problem.
The goals are well defined. The results respond to the objective.
In the method, I suggest mentioning the type of study. The authors present the reference of the original study (reference 21). However, I suggest presenting some details of this study for better understanding.
Variables are clearly described.
The results and discussion are clear.
conclusions that are appropriately drawn based on the data presented.

**RESPONSE:** We added the type of study at the very beginning of the methods.

This is an observational study based on an analysis of survey data collected by the researchers in the early days of the COVID-19 pandemic in the U.S. (lines 118, 119)

**AND**

In sum, this is a convenience sample of English-speaking social media users in the U.S. (lines 128, 129).

Reviewer #2: The manuscript by Capasso et al., seeks to identify the socioeconomic predictors of COVID-19 related health disparities among US workers. The manuscript addresses a major concern as results obtained could be very important in shaping policy. Though the manuscript is properly written and the methods and results properly crafted, they are a few concerns that need to be addressed before the publication.
Listed below are few concerns I have with the manuscript:

1. Generally, I believe that the manuscript needs to be looked at a little more keenly and minor grammatical errors addressed. For instance, the last sentence in line 63 should begin with a conjunction like ‘In additional, moreover, etc. Lines 68 needs to rephrased for clarity. In addition, Line 75 – 76 should also be rephrased.

**RESPONSE:** We carefully read the manuscript to address these issues. We added “In addition,” to the mentioned sentenced (now line 69). We modified line 68 (now lines 73-74) to “COVID-19 affected rural and urban areas in drastically different ways.” We rephrased the sentence in lines 75-76 to:

“Many social and economic factors affected how COVID-19 affected the health and well-being of the U.S. population. These factors were often interconnected and co-occurring, or ‘clustered’ among specific population subgroups.” (new lines 81-82)

2. To provide context, it is important for the abstract to begin with a brief background that highlights the burden of the disease being addressed.

**RESPONSE:** We added the following description to the abstract to provide the context requested:

The COVID-19 pandemic has disproportionately impacted the physical and mental health, and the economic stability, of specific population subgroups in different ways deepening existing disparities. Essential works have faced the greatest exposure risk, women have been burdened by caretaking responsibilities, and rural residents have experienced healthcare access barriers. These factors were often interconnected and co-occurring, or ‘clustered’ among specific population subgroups. While most research has so far focused on individual factors related to COVID-19 disparities, few have explored the complex relationships between the multiple components of COVID-19 vulnerabilities.

3. The abstract lacks the results related to the mental health status of the respondents

**RESPONSE:** We added the following sentence describing the mental health results to the abstract: (lines 45-48)

Women (β = 0.22; B = 0.65, 95% CI = 0.65, 0.74) experienced worse mental health than men; whereas essential workers reported better mental health (β = -0.08; B = -0.25, 95% CI = -0.38, -0.13) than other workers.

4. In the introduction, (line 42 -43), the authors should rather indicate the global burden exerted by COVID-19 and then talk about its impact on the US. The current statement is misleading as it seems to imply that COVID-19 has impacted only the US. In addition, the economic impact mentioned in line 43 is repeated in line 45. This should be corrected.

**RESPONSE:**

We have re-written and consolidated this paragraph, thus (lines 54-60):

The coronavirus disease (COVID-19) pandemic has claimed a devastating toll on people’s physical and mental health [1, 2] and caused social and economic hardship *both globally and* in the United States (U.S.) [3]. In the U.S., surveys have found that 40% of people experienced poor mental health [4], 15% lost their jobs [3], one in seven experienced food insecurity [5, 6], and over 7% of adults experienced housing insecurity [7] during the pandemic.

5. Though the need to provide written or oral consent was waived, the authors should clearly indicate where ethical clearance was obtained from.

**RESPONSE:** The IRB where the ethical clearance was obtained was specified (line 129).
